# Claudins in vulvar cancer – from epithelial barrier to potential tumor-agnostic cancer therapy

**DOI:** 10.1080/21688370.2024.2444724

**Published:** 2024-12-25

**Authors:** Gilbert Georg Klamminger, Annick Bitterlich, Meletios P. Nigdelis, Martin Ertz, Kim Yoo-Jin, Annette Hasenburg, Mathias Wagner

**Affiliations:** a Department of General and Special Pathology, Saarland University (USAAR) and Saarland University Medical Center (UKS), Homburg, Germany; b Department of Obstetrics and Gynecology, University Medical Center of the Johannes Gutenberg University Mainz, Mainz, Germany; c Department of Gynecology, Obstetrics and Reproductive Medicine, Saarland University Medical Center (UKS), Homburg, Germany; d Department of Pathology, Institute for Pathology GbR Kaiserslautern, Kaiserslautern, Germany

**Keywords:** Claudin, claudin 18.2, immunohistochemistry, squamous cell carcinoma, tight junctions, vulvar cancer

## Abstract

The immunohistochemical expression of various members of the claudin family has already been studied in pathological affections of the vulva whether to differentiate precancerous lesions from vulvar squamous cell carcinoma or in inflammatory conditions such as lichen sclerosus. From an oncological perspective, however, immunohistochemical analysis of claudin 18.2 protein expression has become increasingly clinically relevant nowadays since the impressive therapeutic benefits of the claudin 18.2 antibody *zolbetuximab* have been widely recognized. Systematic studies evaluating its expression, including in vulvar cancer, are needed to understand whether such treatment strategies may eventually benefit patients with vulvar neoplasia.

## Claudins in squamous cell carcinoma of the vulva

It was not only due to the scientific paradigm shift away from transcription factors and tyrosine kinases that claudins^1^ – as a component of tight junctions (TJ) not only responsible for paracellular diffusion and the adapting cell membrane permeability but also the most fundamental property of epithelia, namely the cohesion of a cell formation of polar cell forms^2,3^ – became the focus of increasing interest in oncological and histomorphological research. Hereby, especially the claudins 3, 4, 6 and 18.2 have become the main focus of oncological research since their expression across different tumor entities is a necessary prerequisite for a potential tumor-agnostic, pan-cancer treatment within the area of personalized medicine.^4–7^ Scilicet, to date, at least some family members of the total 26 human claudin proteins have been tested in a gyneco-oncological context, whether for the detection of precancerous cervical lesions or the potential risk stratification of solid non-gastric neoplasms.^3,6,8,9^

Regarding the clinical impact of claudin expression even within squamous cell carcinomas of the vulva (VSCC), Sadalla et al. addressed the question of potentially differing expression patterns of certain claudin family members (claudin 1, 2, 3, 4, 5, 7 and 11) in vulvar lesions already in 2011, when examining vulvar samples with lichen sclerosus (LS), isolated squamous cell carcinoma (*n* = 15) and non-affected controls. They determined a loss of claudin 7 and 11 expression and, therefore, a significant difference in protein expression within LS and VSCC in comparison to the respective control group (*p* = 0.013 and *p* = 0.001; chi-square test). Other claudins tested did not show significantly different expression between the groups; within this study cohort, they showed positive expression in VSCC in 66.7% (claudin 3 and claudin 4) to 86.7% (claudin 1).^10^ Following on from this, Riski et al. examined the expression of different claudin members (1, 3 M, 3S, 4, 5 and 7) in precancerous vulvar lesions (vulvar intraepithelial neoplasia, VIN) and VSCC (*n* = 49) taking into account both membranous and cytoplasmatic staining patterns. They reported different staining intensities within neoplastic affections, especially for claudin 3 M in VIN I lesions, VIN II–III lesion and invasive carcinomas (*p* = 0.026; Kruskal–Wallis test) as well as a decreased expression of claudin 3 M in carcinomas compared to low-grade intraepithelial neoplasia (*p* = 0.002; Mann–Whitney test). Although the team of Riski et al. did not determine additional significant differences in claudin expression of other claudin types, they reported an overall positive expression of claudin 7 in the majority of invasive carcinomas – in contrast to the above-mentioned study by Sadalla et al.^10,11^

Recently, the expression of claudin 18.2 (a specific splice variant encoded by the CLDN18 gene) has garnered significant attention and therapeutic relevance – as summarized in a worthwhile article published in *Tissue Barriers* by Kyuno et al.^12^ Phase III studies, such as the SPOTLIGHT and GLOW trials, have demonstrated a potential for extended overall survival and progression-free survival in certain advanced-stage gastric cancers with the administration of the monoclonal anti-claudin 18.2 antibody called *zolbetuximab*.^6,13,14^ Albeit, in physiologic state, claudin 18.2 is almost exclusively expressed within the gastric mucosa, a positive claudin 18.2 expression has also been detected in non-gastric solid tumors such as ovarian cancer and non-small lung cancer, probably due to alterations in the extracellular signal-related kinase and/or protein kinase C pathway.^1^ Before being considered as the next potential tumor-agnostic target/therapy even in additional tumor entities such as VSCC, it is essential to initially examine the immunohistochemical expression of claudin 18.2 in vulvar cancer (VC).

## Immunohistochemical expression of claudin 18.2 in VSCC

**Figure 1.**
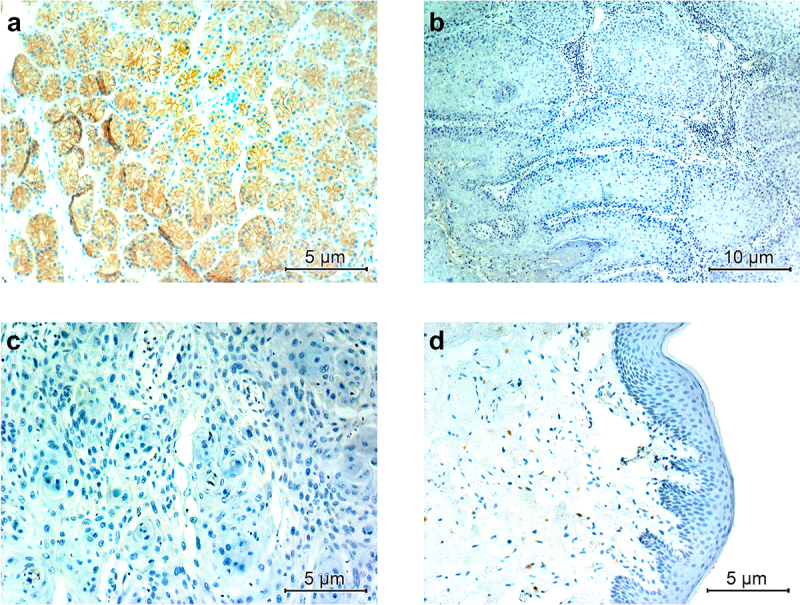
While claudin 18.2 shows distinct (predominantly membranous but also faint cytoplasmatic) staining within gastric mucosa (a), neither squamous cell carcinomas of the vulva (b, c) nor vulvar epithelium (d) in our study cohort immunohistochemically express the tight junction protein of interest.

Within a preliminary analysis, we therefore examined the protein claudin 18.2 expression in 20 VC patients (whole slide analysis), who were diagnosed at the Department of General and Special Pathology, University Hospital of Saarland. HPV (Human Papillomavirus) association was determined according to the diagnostic standards of the current *5th WHO classification of Female Genital Tumors* using a routine p16 (CINtec® p16, Roche, Switzerland) antibody and protocol. Hereby, ‘blocktype’ staining of at least 20 neighboring neoplastic cells was considered as positive.^15^ In total, ten HPV-associated tumors and ten HPV-independent tumors were tested, taking into account the potential HPV-dependent oncogenesis and the resulting distinct tumor entities (HPV-associated VC and HPV-independent VC). Within our patient cohort, the median age was 55.5 years; classified by the TNM (TNM Classification of Malignant Tumors) stage (8th edition), five patients had pT1a tumors, ten patients had pT1b tumors, and five patients had T2 tumors. Table 1 depicts the clinico-pathological tumor characteristics of the examined tumors in detail. Despite the evaluation of claudin 18.2 expression within the neoplastic cells, its potential expression in regular vulvar squamous epithelium, which could be found in the immediate proximity of the carcinoma and any potential pre-cancerous lesions, was also assessed wherever feasible. All conducted experiments were approved by the Ethics Committee of the Saarland Medical Association (249/23); data handling was further in best alignment with the Declaration of Helsinki.^16^ Tissue samples were prepared for standard immunohistochemical analysis using a commercially available anti-claudin 18.2 antibody and protocol (DCS Diagnostics, Hamburg, Germany); the chromogen diaminobenzidine was employed. Non-neoplastic gastric mucosa served as an on-slide positive control (Figure 1(a)); additional on-slide negative controls of claudin 18.2 negative pancreatic adenocarcinomas confirmed specificity.

**Table 1.** Pathological information about the tumor samples within our selected cohort. We included patients with different tumor stages and varying groin lymph node status but also all four grades of tumor differentiation (Broder’s grading I–IV) and HPV association (p16).

Sample number	T-stage	Depth of infiltration in cm	N-stage	V	Pn	L	Grading (Broder’s grading)	p16 IHC
1	T1a	0.06	0	No	No	No	Grade I	Negative
2	T1b	1.8	0	No	No	No	Grade III	Negative
3	T1b	0.4	0	No	No	No	Grade III	Negative
4	T2	1.5	0	No	No	No	Grade II	Negative
5	T1a	0.03	0	No	No	No	Grade III	Negative
6	T1b	1.7	1a	Yes	Yes	Yes	Grade II	Negative
7	T1a	0.05	0	No	No	No	Grade I	Positive
8	T1b	0.2	0	No	No	No	Grade I	Negative
9	T1b	0.5	0	No	No	No	Grade I	Positive
10	T1b	0.4	0	No	No	No	Grade I	Negative
11	T2	1.9	2c	Yes	No	Yes	Grade IV	Negative
12	T1b	1.1	2c	No	No	Yes	Grade I	Negative
13	T1b	0.4	0	No	No	No	Grade I	Positive
14	T1b	0.2	0	No	No	No	Grade IV	Positive
15	T1a	0.05	0	No	No	No	Grade III	Positive
16	T2	>0.1	0	No	No	No	Grade II	Positive
17	T1b	0.4	0	No	No	No	Grade I	Positive
18	T2	3.5	2c	No	No	No	Grade I	Positive
19	T1a	0.05	0	No	No	No	Grade I	Positive
20	T2	0.4	0	No	No	No	Grade I	Positive

All our examined VSCC samples did not show any immunohistochemical expression of claudin 18.2 – regardless of clinical stage, tumor differentiation (histological grading) or oncogenesis (HPV status), see Figure 1(b,c). Furthermore, the physiological vulvar epithelium demonstrated an absence of claudin 18.2 expression of all epithelial layers (Figure 1(d)).

## Future perspective

As an exploratory experiment, we tested a small cohort of VSCC with regard to the immunohistochemical expression of claudin 18.2 and did not detect any such expression. Albeit with the advantage of a whole slide analysis performed, our potentially treatment-relevant findings of a small cohort must remain preliminary. Future studies, sufficiently powered to detect even potential low overall incidences of claudin 18.2 expression, could be performed using a resource-efficient approach such as the employment of tissue microarrays, which allows for the implementation of a high number of tumor samples with the combined advantages of less antibody usage. A distinct indication to conclude a heterogeneous protein expression of claudin 18.2 in VC and therefore to prefer a whole slide analysis even with larger numbers of cases cannot be derived from our data presented. From a clinical perspective, potential targeting of claudin 18.2 in VC could highlight the ongoing transition to a more individualized and molecular-based oncological therapy even in rare tumors such as VC, since tumor-agnostic therapies (which allow for tumor entity-independent treatment solely based on distinct molecular biological cancer features) have yet already been implanted within common treatment algorithms of VC. According to the current *NCCN clinical practice guideline* (*Vulvar Cancer, Version 3.2024*), e.g., NTRK gene fusions – which occur in about 0.3% of solid tumors^17^ – could qualify for TRK inhibitor (*entrectinib* and *larotrectinib*) involving second/third-line therapy.^18^ As with claudin 18.2, the first step here is immunohistochemical testing of an FFPE tumor block to evaluate the biological plausibility and consecutive possibility of a targeted treatment strategy in general. However, distinct cutoff values for claudin 18.2, which would constitute clear therapeutic advantages, have not yet been established, and current approaches favor at least >75% moderate-to-strong stained cancer cells of a lesion to be considered as ‘claudin 18.2 positive’. In contrast to NTRK gene fusions, a positive claudin 18.2 immunohistochemistry does not require affirmative molecular testing.

## Conclusion

In summary, histological research on claudins/tight junctions has recently undergone a broadening of perspective – from fundamental questions about the pathobiological ultrastructure of epithelial tumors to potentially therapy-relevant biomarkers. It is now the systematic evaluation of its immunohistochemical expression in solid epithelial tumors such as VSCC, which will ultimately identify patients with potential clinical benefits also besides current standards.

## References

[1] Nakayama I, Qi C, Chen Y, Nakamura Y, Shen L, Shitara K. Claudin 18.2 as a novel therapeutic target. Nat Rev Clin Oncol. 2024;21(5):1–5. doi:10.1038/s41571-024-00874-2. 38503878

[2] Tsukita S, Yamazaki Y, Katsuno T, Tamura A, Tsukita S. Tight junction-based epithelial microenvironment and cell proliferation. Oncogene. 2008;27(55):6930–6938. doi:10.1038/onc.2008.344. 19029935

[3] Günzel D, Yu ASL. Claudins and the modulation of tight junction permeability. Physiol Rev. 2013;93(2):525–569. doi:10.1152/physrev.00019.2012. 23589827 PMC3768107

[4] Mackensen A, Haanen JBAG, Koenecke C, Alsdorf W, Wagner-Drouet E, Borchmann P, Heudobler D, Ferstl B, Klobuch S, Bokemeyer C, et al. CLDN6-specific CAR-T cells plus amplifying RNA vaccine in relapsed or refractory solid tumors: the phase 1 BNT211-01 trial. Nat Med. 2023;29(11):2844–2853. doi:10.1038/s41591-023-02612-0. 37872225 PMC10667102

[5] McDermott MSJ, O’Brien NA, Hoffstrom B, Gong K, Lu M, Zhang J, Luo T, Liang M, Jia W, Hong JJ, et al. Preclinical efficacy of the antibody–drug conjugate CLDN6–23-ADC for the treatment of CLDN6-positive solid tumors. Clin Cancer Res. 2023;29(11):2131–2143. doi:10.1158/1078-0432.CCR-22-2981. 36884217 PMC10233360

[6] Sahin U, Koslowski M, Dhaene K, Usener D, Brandenburg G, Seitz G, Huber C, Türeci O. Claudin-18 splice variant 2 is a pan-cancer target suitable for therapeutic antibody development. Clin Cancer Res. 2008;14(23):7624–7634. doi:10.1158/1078-0432.CCR-08-1547. 19047087

[7] Hashimoto Y, Yagi K, Kondoh M. Current progress in a second-generation claudin binder, anti-claudin antibody, for clinical applications. Drug Discov Today. 2016;21(10):1711–1718. doi:10.1016/j.drudis.2016.07.004. 27422269

[8] Sobel G, Páska C, Szabó I, Kiss A, Kádár A, Schaff Z. Increased expression of claudins in cervical squamous intraepithelial neoplasia and invasive carcinoma. Hum Pathol. 2005;36(2):162–169. doi:10.1016/j.humpath.2004.12.001. 15754293

[9] Rahman A, Kobayashi M, Sugimoto K, Endo Y, Kojima M, Furukawa S, Watanabe T, Soeda S, Hashimoto Y, Fujimori K, et al. Reduced claudin-12 expression predicts poor prognosis in cervical cancer. Int J Mol Sci. 2021;22(7):3774. doi:10.3390/ijms22073774. 33917356 PMC8038723

[10] Sadalla JC, Lourenço SV, Sotto MN, Baracat EC, Carvalho JP. Claudin and P53 expression in vulvar lichen sclerosus and squamous-cell carcinoma. J Clin Pathol. 2011;64(10):853–857. doi:10.1136/jclinpath-2011-200103. 21642657

[11] Riski M, Santala M, Soini Y, Talvensaari-Mattila A. Claudins 1, 3M, 3S, 4, 5 and 7 in vulvar neoplasms compared with vulvar squamous cell carcinoma. Tumor Biol. 2012;33(33):537–542. doi:10.1007/s13277-011-0289-8. 22170432

[12] Kyuno D, Takasawa A, Takasawa K, Ono Y, Aoyama T, Magara K, Nakamori Y, Takemasa I, Osanai M. Claudin-18.2 as a therapeutic target in cancers: cumulative findings from basic research and clinical trials. Tissue Barriers. 2022;10(1). doi:10.1080/21688370.2021.1967080. PMC8794250 34486479

[13] Shah MA, Shitara K, Ajani JA, Bang Y-J, Enzinger P, Ilson D, Lordick F, Van Cutsem E, Gallego Plazas J, Huang J, et al. Zolbetuximab plus CAPOX in CLDN18.2-positive gastric or gastroesophageal junction adenocarcinoma: the randomized, phase 3 GLOW trial. Nat Med. 2023;29(8):2133–2141. doi:10.1038/s41591-023-02465-7. 37524953 PMC10427418

[14] Shitara K, Lordick F, Bang Y-J, Enzinger P, Ilson D, Shah MA, Van Cutsem E, Xu R-H, Aprile G, Xu J, et al. Zolbetuximab plus MFOLFOX6 in patients with CLDN18.2-positive, HER2-negative, untreated, locally advanced unresectable or metastatic gastric or gastro-oesophageal junction adenocarcinoma (SPOTLIGHT): a multicentre, randomised, double-blind, phase 3 trial. The Lancet. 2023;401(10389):1655–1668. doi:10.1016/S0140-6736(23)00620-7. 37068504

[15] WHO Classification of Tumours Editorial Board. WHO Classification of Tumours Series. Female Genital Tumours. Vol. 4. 5th ed. Lyon, France: International Agency for Research on Cancer; 2020.

[16] World Medical Association Declaration of Helsinki. JAMA. 2013;310(20):2191. doi:10.1001/jama.2013.281053. 24141714

[17] Doebele RC, Drilon A, Paz-Ares L, Siena S, Shaw AT, Farago AF, Blakely CM, Seto T, Cho BC, Tosi D, et al. Entrectinib in patients with advanced or metastatic NTRK fusion-positive solid tumours: integrated analysis of three phase 1–2 trials. Lancet Oncol. 2020;21(2):271–282. doi:10.1016/S1470-2045(19)30691-6. 31838007 PMC7461630

[18] Abu-Rustum NR, Yashar CM, Arend R, Barber E, Bradley K, Brooks R, Campos SM, Chino J, Chon HS, Crispens MA, et al. Vulvar cancer, version 3.2024, NCCN clinical practice guidelines in oncology. J Natl Compr Cancer Network. 2024;22(2):117–135. doi:10.6004/jnccn.2024.0013. 38503056

